# The experience of caregivers providing therapeutic patient education for people living with bipolar disorder: a qualitative study

**DOI:** 10.1186/s12888-023-04623-0

**Published:** 2023-03-24

**Authors:** Marion Chirio-Espitalier, Yves-Antoine Harscoët, Mélanie Duval, Julien Jupille, Leïla Moret, Marie Grall-Bronnec

**Affiliations:** 1 Psychiatry and Mental Health Department, University Hospital of Nantes, 85 Rue Saint-Jacques, 44093 Nantes Cedex 1, France; 2 UMR INSERM U1246-SPHERE ‘methodS for Patients-Centered Outcomes & HEalth REsearch’, University of Nantes, University of Tours, 22 Boulevard Benoni Goullin, 44000 Nantes, France; 3 CRehab’S, Support Center for Psychosocial Rehabilitation in the Loire Region, Nantes University Hospital and Angevin Mental Health Center, Nantes, France; 4 Public Health Department, University Hospital of Nantes, 85 Rue Saint-Jacques, 44093 Nantes Cedex 1, France

**Keywords:** Bipolar disorder, Therapeutic patient education, Therapist experience, Caregiver posture, Personal recovery, Qualitative research

## Abstract

**Background:**

Therapeutic patient education (TPE) programs are psycho-educational treatments suggested for all chronic diseases. For several years, these programs have been developing for people living with bipolar disorder. However, to date, only few qualitative studies have explored the experience of caregivers. We wanted to explore the experience of caregivers working in psychiatry as facilitators of a therapeutic education program for people living with bipolar disorder.

**Method:**

A single-center qualitative study was carried out. We conducted an inductive exploration, examining the content of the discourse produced in a focus group of eight caregivers in therapeutic education. The corpus was transcribed manually and a thematic analysis was conducted by two authors in a blinded fashion before combining.

**Results:**

Four dimensions and twenty themes were identified: i) facilitators' pleasant experiences of the TPE sessions with a secure climate and a sense of belonging to a group, ii) being a TPE facilitator with a new horizontal and collaborative posture valuing the experiential knowledge, iii) the role of the TPE sessions with knowledge provision, empowerment and destigmatization, and iv) perceived changes in patients with an appeasement, the awareness of a shared experience, openness to others, a phenomenon of identification to peers and a new commitment.

**Conclusions and implications for practice:**

The observations noted overlap with the elements of the personal recovery well known CHIME framework (Connectedness, Hope, positive Identity, Meaning in life and Empowerment). Therapeutic education is a developing form of psychosocial rehabilitation care: through the mobilization of a new attitude of caring, the facilitation of TPE programs could be a lever for changing the posture of caregivers in favor of supporting the personal recovery of people with bipolar disorder. These results would need to be confirmed by further studies.

## Impact and implications

The caregivers interviewed who lead therapeutic education groups described a change in their caregiving posture, which was more horizontal and collaborative. Therapeutic education thus fostered empowerment and destigmatization, promoting patients appeasement and openness. The observations overlap with personal recovery levers. Facilitation of therapeutic education could help caregivers move towards a more recovery-oriented care posture.

## Introduction

The World Health Organization considers bipolar disorder (BD) to be one of the ten most disabling chronic pathologies [1], and it has a prevalence of 2.6% in the general population [2, 3]. Frequent relapses in bipolar disorder have serious impacts on the psychosocial functioning, cognition, quality of life, and survival of affected individuals [4, 5]. Management recommendations for BD [6–8] call for a combination of a pharmacological approach and psychosocial interventions. Among the latter, there is a good level of evidence for psychoeducation [7, 9]. Psychoeducation is an individual or group-based intervention that aims to provide knowledge and skills to individuals affected by a chronic psychiatric disorder so that they can better manage their lives.

Quantitative studies have indicated that psychoeducation has a beneficial impact on BD by reducing the rate of relapse, increasing the time between episodes of decompensation, decreasing the number and length of hospitalizations [10–12], and improving patients' treatment adherence and social functioning [13–15]. This therapeutic approach is considered promising for other psychiatric disorders such as schizophrenia. Its favourable effects were found in the field of schizophrenia, as shown by two systematic reviews [16, 17]. Psychoeducation has been shown to be effective in reducing relapse, length of hospitalization and medication compliance [16]. Although the level of evidence is still modest for brief programs, there is a short-term effect on the number of relapses and a medium-term effect on medication compliance [17].

In France, the “Hospital, Patients, Health and Territories” Act of 2009 included therapeutic patient education (TPE) in the Public Health Code [18] and thereby established a regulatory framework for TPE. Access to TPE programs is recommended for any chronic disease. TPE *"aims to help patients acquire or maintain the skills they need to manage their lives with a chronic disease"* [19] and must be adapted to the specific needs of each participant via an *"educational diagnosis"* evaluation. This degree of personalization in TPE differs from that in psychoeducation programs. The regulatory framework recommends the co-construction and co-facilitation of TPE programs with people concerned about the specific disease.

Only a few studies have explored the experience of caregivers as facilitators in TPE. In the field of psychiatry, there are no articles to our knowledge that have explored the experiences of the facilitators of these programs. A recent study was interested in the representations of caregivers concerning the practice of TPE, and highlighted the great reluctance to use this care tool, which would conflict with a traditional model more paternalistic than collaborative [20]. The aim of our work was therefore to qualitatively explore the experience of caregivers and peer helpers working in psychiatry as facilitators of a TPE program for people living with BD. Our intention was to describe what happens during TPE sessions from the facilitators' point of view and what they believe could promote the well-being and recovery process of people living with BD.

### The therapeutic patient education program

#### Program construction and implementation

The studied TPE program was dedicated to people living with BD and was developed by the research team in 2016; the team developed the program according to the national recommendations in force (HAS, 2007). The program has since been facilitated by the professionals of the Psychosocial Rehabilitation Center (PSR): nurses, peer helpers, and psychiatric physicians. It had been running on a regular basis for 5 years when the study was conducted.

#### Program progress

The program was co-facilitated by two to three health professionals and peer helpers trained in TPE. Each TPE group included 10 patients. It was conducted following four stages: i) an individual assessment interview, leading to an “educational diagnosis”; ii) nine weekly group sessions lasting two hours (except for the eighth session, which lasted three hours); iii) a final individual interview to evaluate the skills developed by the participants and their satisfaction with the program; and iv) a group consolidation session three months after the ninth session.

#### Description of the program sessions

Each session began with an "ice-breaker", followed by a debriefing on the previous session using participatory animation tools. A theme was addressed during the first hour; after a short break, a second theme was addressed, and the session ended with a period for synthesis and a mood self-assessment. An individual time could be proposed to participants who felt the need. The team created a booklet that followed the themes of the program that was given to the participants so that they could write down information throughout the sessions.

Table 1 presents the themes addressed and the objectives of the sessions.

**Table 1:** Description of the TPE bipolar disorders program sessions

Session and duration	Theme covered	Objectives
Session 1, 2 h	Collective educational diagnosis	Create a safe and caring environment Cocreate rules of operation Specify the modalities of a TPE program Define and share the priority themes to be explored according to the participants' needs
Session 2, 2 h	Bipolar disorders	Define bipolar disorders Understand the risk factors
Session 3, 2 h	Symptomatology, risk factors and protective factors	Identify risk factors and protective factors Identify symptomatology Understand how to prevent suicidal risk
Session 4, 2 h	Treatments	Deepen knowledge of drug and nondrug treatments
Session 5, 2 h	Taking care of oneself	Learn to take care of one’s mental health Develop coping skills Identify activities and determinants that promote mental health
Session 6, 2 h	Preventing relapse	Identify the prodromes Increase awareness of the joint crisis plan
Session 7, 2 h	Preparing for the session with family and friends	Define the topics to be covered according to the needs of the participants in session 8
Session 8, 3 h	Session with loved ones	Determine the topics to be discussed Talk about the disease with family and friends Define joint actions to prevent relapses
Session 9, 2 h	Debriefing session	Evaluate participation in the program
Consolidation session at 3 months, 2 h	Consolidation session	Consolidate participants' skills and knowledge

## Method

### Type of research

Our study was based on an inductive and monocentric qualitative exploratory methodology. For this purpose, we conducted one focus group (FG) of health professionals (nurses, psychiatrists and psychiatric residents) and peer helpers facilitating a TPE program for bipolar disorders in a psychosocial rehabilitation center located in a Psychiatry and Mental Health Department. This FG was conducted in January 2021. The framework of the FG was developed by a public health intern and a psychiatric physician who facilitated the FG and were not involved in the care. The themes that were addressed following a semidirective guide were i) the facilitators' motivation to facilitate a TPE program; ii) representations of the role of the TPE facilitators; iii) caregivers' experiences; iv) changes observed in the participants by the facilitators; and v) dimensions associated with the observed changes.

### Study population

Our sample targeted the study population of health professionals and expert patients running the "Bipolar Disorders" TPE program. The inclusion criteria for this study were i) to have facilitated at least one session of the bipolar disorders TPE program studied in the past year; ii) to have received 40 h of academic training in the facilitation of TPE programs; and iii) to be available for the duration of the FG.

### Mode of collection

After receiving the agreement of the head of the PSR center, we presented our study project to the health care team in charge of running the "Bipolar Disorders" TPE program. At the time of the study, two sessions of the program were taking place in parallel, with each program having its own team of facilitators. We offered all the TPE program facilitators the opportunity to participate in the FG, and all agreed. A single FG was convened with all facilitators from the ongoing programs. The FG, which lasted one and a half hours, was facilitated by two health professionals, a public health intern trained in FG facilitation and a psychiatric physician; the latter were not involved in facilitating the TPE program. Two observers were also present: a public health intern not involved in the program, and an advanced practice nurse who was familiar with the program from having previously co-facilitated it. The observers wrote down the participants' reactions and responses. With the participants' consent, an audio recording was made and stored on a password-protected audio file. Transcription was performed manually without software support. The recordings were deleted once they were transcribed. The sociodemographic data of the sample were also collected during the FG.

### Method of analysis

The FG corpus was transcribed manually by the first author. To process the data, we relied on the method of thematic analysis [21] which consisted of capturing all of the themes of the data collected during the FG to identify the central themes that answered the research question. This method was composed of the following steps: i) coding/thematization; ii) categorization; iii) linking of the data; iv) presentation of the data; and v) verification of the data.

This method made it possible to understand and interpret the data while remaining faithful to what the participants said. The reading of the FG transcript and its analysis were carried out by an advanced practice nurse and a psychiatric physician in a blinded fashion. Neither of these two individuals had been involved in the facilitation of this program under review, but they had previous experience in facilitating it. A consultation between these two authors took place at the end of the coding process to compare the themes identified by each and to create links between the categories. All themes were identified by both researchers, and agreement between them on the organization of the categories was reached.

### Ethical considerations

Each participant was given an oral presentation of the study design, and signed informed consent was obtained. The protocol was validated by the ethics committee (Groupe Nantais d'Ethique dans le Domaine de la Santé, GNEDS) on 23 July 2020. To avoid the identification of any participant, the data collected in this study were processed in an entirely anonymous manner. Anonymity was ensured by a numerical code accessible only by the researchers.

## Results

In this study, our sample consisted of eight facilitators from the TPE program studied.

### Simple description of sociodemographic data

The average age of the participants was 40.5 years; the minimum age was 29 years, and the maximum age was 66 years. Half of the participants were between 33 and 49 years of age, 3 were between 18 and 33 years of age, and 1 was over 50 years of age. The standard deviation of ages was 11.32, and the median age was 37.5 years. The majority of the participants were women (*N* = 6).

Of the eight facilitators, two were psychiatric physicians (PPs), two were state-registered nurses (Ns), two were volunteer peer helpers (VPHs) (one retired, one a craftsman), one was a psychiatric intern (PI), and one was a hospital-employed peer health mediator (EPH). All had experience working in a psychosocial rehabilitation department.

Experience in facilitating TPE sessions ranged from less than 5 sessions facilitated (*N* = 1) to more than 30 sessions facilitated (*N* = 1). More than half of the facilitators had facilitated at least eleven TPE sessions (*N* = 6). Two-thirds of the co-facilitators (*N* = 6) had participated in the facilitation of at least four different sessions of the studied TPE program; two of these facilitators had co-facilitated the program four times, two six times, one seven times and one eight times. The other two had co-facilitated the TPE program one and twice.

### Discourse analysis

Four dimensions were identified during the qualitative analysis of the corpus: i) facilitators' experiences of the TPE sessions, ii) being a TPE facilitator, iii) the role of the TPE sessions, and iv) perceived changes in patients.

For each dimension, themes and subthemes emerged. A summary of the results is presented in Table 2. The detailed presentation of the results of the discourse analysis is presented next; the participants are identified anonymously with the following information: role, gender, and age.

**Table 2:** Dimensions, themes and subthemes from the corpus

Dimensions	Themes	Subthemes
Facilitators' experiences of TPE sessions	Climate of security	Feeling confident
		Allowing oneself to be
		Sense of freedom
		Caring
		Connecting participants
	Belonging to a group	Belonging to a team of facilitators of TPE sessions
		Belonging to "something I believe in”
	Experience sharing	Peers: sharing their experience
		Caregivers: contributing to experience sharing
	Enjoyment of facilitation	
Being a TPE facilitator	An expert	Expert by experience
		Medical expert
	Peer helpers	A bearer of hope
		A benchmark
	Guarantor of the TPE framework	
	Horizontal relationship	Natural posture
		“Equal to equal”
		Participate in exchanges, self-disclosure
	Listening position	
	Educational posture	
The role of TPE sessions	Contribution of knowledge	Learning from experience
		Learning space for caregivers
	Promoting empowerment	Giving freedom
		Giving autonomy
	Self-normativity Destigmatisation	Self-awareness
		Building recovery on one's own standards
The changes perceived by the FG participants in patients who have used the TPE program	Appeasement	Through destigmatization
	Awareness of a common experience	
	Openness	To one’s peers
		To one’s loved ones
		To the breaking down of taboos
		To psychiatrists
	Identification	With other participants
		With the peer helper
	Quality of life	
	First stage of re-engagement	Sense of self-efficacy
		Self-esteem
	Grief	

### Facilitators' experiences of TPE sessions

This dimension was associated with four themes: a climate of safety, belonging to a group, sharing experiences and the pleasure of facilitation. The theme of safety was associated with three subthemes: feeling confident, allowing oneself to be authentic, and the notion of caring. One of the main elements in providing a safe climate was the desire to create a space of trust:

"It is my desire to facilitate a climate of authenticity and listening in the group" (EPH, female, 33).

One of the facilitators noted what a patient said about being listened to:

"It reminds me of what a patient said at one point, as if it was very surprising. He said, 'It's incredible here they listen to us,' as if in a psychiatric hospital, you can't be listened to. He couldn't believe it. It was quite spectacular to hear this come from his own mouth" (VPH, male, 66 years old).

The notion of authenticity was taken up and explained in these terms:

"To have an identity that is my own, that can be different from others" (EPH, female, 45 years old). One facilitator mentioned the desire to "create this space where (…) he feeds both the feeling of freedom and the need for relationship" (PP, female, 32 years old).

This intention for relationships and connection was taken up again by another participant:

"A good atmosphere and to make sure that there is a link between people, that there is exchange, and that people can have moments of listening with their peers that they don't necessarily have outside the sessions, of support in fact from others, to develop a feeling of belonging, to live better with it afterward" (N, female, 44 years old).

Creating this space of security was reinforced by the notion of benevolence.

"which is quite present in TPE and which is good for the group and good for ourselves" (PI, male, 29 years old).

This benevolence created a climate of security for the participants and the facilitators:

"There is something benevolent in the group in general, which makes me feel good" (N, female, 42 years old).

Half of the facilitators interviewed (*N* = 4) mentioned the feeling of belonging to a group shared with the users:

"It is first of all that we are in a group, that the patients I meet in another context than the crisis" (N, female, 42 years old); "to be part of this adventure, which is something I believe in, which is everything that is mutual aid groups" (VPH, female, 45 years old).

The majority of the facilitators (*N* = 7) verbalized the feeling of belonging with colleagues and associated it with a notion of pleasure:

"One of my motivations for participating is already to find people from the service; being able to lead the groups is always a pleasure for me" (PI, male, 29 years old).

For one of the facilitators, the fact that the program was run in a group setting encouraged the sharing of experiences:

"The idea that they are in a group, that they share with their peers… The sharing of experiences seems to me to be very rich" (PP, female, 32 years old).

This point was supported by other participants:

"There is this sharing between peers, which is very important. It is very interesting because it leaves room for knowledge through experience" (PP, female, 32 years old).

Others mentioned the effects of this sharing, stating that

"these TPE groups allow for an exchange between participants and an awareness of certain symptoms or disorders" (PI, male, 29 years old) and adding that "there is also the fact that exchanging between peers allows some people to get out of the denial in which they are to varying degrees" (VPH, male, 66 years old).

All the participants in the FG expressed their pleasure in leading these TPE sessions:

"It feels good in general. After a TPE session, I feel good" (PI, male, 29 years old).

### Being a facilitator of TPE sessions

This dimension highlighted the following themes: the place of peer helpers, having an expert role, taking an educational posture, taking a listening posture, having a horizontal relationship and being the guarantor of the framework. The place of peer helpers was associated with being.

*"a reference point. That's what I felt when I had a peer helper as a facilitator, that he was a pillar of recovery"* (VPH, female, 45 years old).

For this facilitator, embodying.

"a witnessing role (…) of a patient with bipolar disorder in recovery (…) that exists, that you can see" meant being a bearer of hope toward a recovery process.

The lived experience of the illness was also associated with an expert role. One of the peer-helper facilitators described his role as follows:

"When I am in the TPEs, it is to validate certain questions (asked by the participants in the TPE program) with my experience, my experimentation. It gives legitimacy to a whole bunch of sometimes quite delirious things that we may have experienced but that exist" (VPH, female, 45 years old).

This function of expert of experience was contrasted with the role of medical expertise. One of the participating doctors showed his discomfort with.

"delivering medical information. I feel like I'm taking on a bit of a posture (…); like the doctor who is a guest on a TV show, ‘So doctor, tell us’ (laughter). At the same time, I feel that sometimes, there are questions that need to be answered and that we have some answers, and I want to pass on knowledge and information, and it can be nice to have this medical hat on" (PP, female, 32 years old).

One of the psychiatrists involved in TPE mentioned that the expert posture could give way to an educational posture:

"When I am a facilitator, I feel that I am leaving my medical and caregiving posture to be more an educator. I have that feeling. I am trying to create a climate conducive to learning by transmitting information" (PP, female, 32 years old).

Another facilitator emphasized being in a listening posture:

"During the TPE groups, I spend a lot of time in a listening position. Everyone around the table, I feel like I am listening to them" (PP, female, 32 years old).

Most of the facilitators had perceived a tendency to be in a horizontal posture of collaborative partnership in interaction with their co-facilitators and with patients:

"What we aim for in TPE is horizontality" (PP, female, 32 years old);"It's about being on the same level in fact" (VPH, female, 45 years old).

Finally, one of the psychiatrists mentioned the importance of being a guarantor by ensuring.

"the notion of a framework, the organization of the session, paying attention to time, sharing the word (…) The framework and the flexibility in the framework" (PP, female, 32 years old).

### The role of TPE sessions

The following three themes were used to clarify this dimension: knowledge provision, empowerment, self-normativity and destigmatization. The TPE facilitators mentioned the contribution of knowledge both for themselves and for the patients through exchanges:

"I am almost in the position of the one who is also learning, who is learning, who is growing at the same time as the group" (N, female, 42 years old). Another added "… it taught me a lot of things in fact. I think it improves my daily practice and the support I can offer my patients" (PP, female, 32 years old).

Others underscored the pleasure of.

"being able to accompany, encourage, transmit knowledge" (VPH, woman, 45 years old) and "to bring clarity to people concerned by psychological disorders, on the disorders themselves" (PP, woman, 32 years old).

Associated with this theme was the promotion of empowerment:

"To create this space where we tell them that there is another possible way than the one of dependence on care, to find their own resources, to better understand themselves" (PP, female, 32 years old).

This notion of empowerment could also be promoted through experiential sharing:

"We can explain things, but it won't have the same impact as someone who has experienced the same disorders or who has gone through certain things or who has taken certain treatments, who has realized that with certain treatments, it was effective or not" (PI, male, 29 years old).

The notion of empowerment could also be promoted through the provision of tools to develop autonomy:

"I like this image of the toolbox, that they can leave at the end with a toolbox of the TPE session, that they can choose the tools they prefer to work with" (N, female, 44 years old).

For some participants, the role of TPE sessions was to promote self-normativity by helping them.

"to really find their own standards within themselves by trying to get away from the injunctions of the outside world, of society, to really build their recovery on who they are, on their needs" (EPH, female, 33 years old).

For others, the role of TPE sessions was to promote destigmatization by talking about.

"the illness, in general, which is quite stigmatized. For personal reasons, I really want to be able to put words to it, to have things said, to be able to put words to it" (PP, female, 32 years old).

### Changes perceived by the TPE facilitators in patients who had used the TPE program

This dimension included seven themes: relief, awareness of a shared experience, openness, identification, quality of life, a first stage of re-engagement, and grief. The notion of appeasement was the first change identified in patients; this change emerged through the destigmatization of words and experiences:

"It's about appeasement. The fact of being able to verbalize in the group. The fact that it can be heard by others, that it is not judged, accepted, validated because we have gone through the same things" (N, female, 42 years old).

Another change observed was awareness, which is an internal process of transformation that changes representations of the self and the world. One caregiver observed among patients:

"the awareness that there is a common experience of the disorder" (N, female, 44 years old).

This awareness may also have had a negative effect. As one participant described, the awareness.

"of one's real limitations, that's something that can seem negative in TPE; patients are all at different stages of their disease, not necessarily all the same" (VPH, female, 45).

This TPE facilitator verbalized that she observed a grieving process among the patients, understood here as a cognitive and affective process that accompanies the loss of someone or something:

"the different phases of grief; in any process there is grief. What's funny is that when you're in this TPE, they're all addressed together. There are some who are in acceptance, recovery (…). A patient was quite in denial at the beginning and all that. It progresses; it progresses, and finally, all the phases of mourning are approached. Depending on where each person is at, they have the possibility of moving on to the next phase; there is a process that takes place" (VPH, woman, 45 years old).

However, the phenomenon of identification with peers, and in particular with the peer helper, could make it possible to overcome this negative effect:

"People identify with the person who has a pathology and who has recovered. They say to themselves "why not me?" False shame disappears; for example, when you start talking about your delusions, "he too", this necessarily breaks down barriers. The peer identifies with the participants who are also there. Double movement. It is because there is a double identification that complex exchanges take place and everyone identifies" (VPH, male, 66 years old).

The FG participants also perceived a change in openness on the part of the patients. This openness was first described toward their peers:

"As the sessions progress, participants will allow themselves to say things that they have experienced or gone through because they know that it will be heard and validated, because it will be experienced or in any case welcomed without being stigmatized" (N, female, 42); "at the end of the TPE, there is often a group created on WhatsApp® to keep in touch; they have become aware that it is a key to recovery to be supported by peers" (VPH, female, 45).

For one participant, one of the effects of openness was the destigmatization:

"(…) Taboos that disintegrate. There are people who say 'if he or she talks about it, I can talk about it too'. Under certain conditions of prudence, it can be an opportunity to talk about it with those close to them. It frees up speech" (VPH, male, 66 years old).

This transition toward openness toward loved ones was also highlighted by another participant:

"In the session with the loved ones, accepting that what you do as work is shared with the most intimate people and that it succeeds like the ones we have been able to live that pffff, (sic.)… it mobilizes a lot of things" (VPH, female, 45).

Another participant identified an openness toward psychiatrists via.

"a kind of destigmatization of psychiatrists (…) they are also there to help people" (PI, male, 29 years old).

This interviewee also suggested an impact on the quality of life of the patients, defined by the World Health Organization (WHO) as.

“individuals' perception of their position in life in the context of the culture and value systems in which they live and in relation to their goals, expectations, standards and concerns”. [22]:"With (…) the toolbox, we can manage daily life a little bit, improve in the end to have a more fulfilled life with what we have seen in TPE" (PI, male, 29 years old).

The notion of commitment was mentioned:

"Participating in the TPE group is a first step to get back into action, to take care of oneself again" (PP, female, 32 years old).

This empowerment was underlined through this example:

"A participant was able to build her own information booklet based on her skills and transmit them to the whole group, and that was very important for her and useful for the other participants" (PP, female, 32 years old).

This action reinforced the participant’s feeling of personal efficiency in managing her disease in her daily life.

## Discussion

### General discussion

#### Main results

Our study examined the experiences of caregiver facilitators of TPE groups in the specific area of bipolar disorder in psychiatry. The facilitators emphasized that their particular posture in the TPE program involved horizontality, a feeling of belonging to a common group with the participating patients, the sharing of experiences and even self-disclosure. They also said that they observed the effects of these programs on the participants; in addition to mutual knowledge, they insisted on the notions of empowerment and destigmatization. Mutual identification with peer helpers seemed to favor identity reconstruction, or at least a form of appeasement.

### TPE and the personal recovery process

Personal recovery from psychiatric disorders is a journey toward a satisfying and hopeful life despite the possible persistence of symptoms. It is described as.

“a deeply personal, unique process of changing one’s attitudes, values, feelings, goals, skills and/or roles… a way of living a satisfying, hopeful and contributing life even with the limitations caused by illness” [23].

The CHIME framework was developed based on a literature review conducted by Leamy and her colleagues [24], who identified five processes as important factors for recovery: connectedness, hope and optimism about the future, identity, meaning in life, and empowerment. In our study, some of the dimensions reported by facilitators appear to correlate with those of recovery in the CHIME model. i) The connectedness dimension seems to be found in the connection to others and in particular to peers – both fostered by the soothing and the source of soothing – based on the feeling of belonging to a group of peers and the support provided by them. ii) The hope dimension is represented by the presence of peers in the recovery process and fostered by the sharing of tools that enable people to manage their daily lives and thus have a more fulfilling outlook on life. iii) The identity dimension is present through the notions of destigmatization and the sharing of experiences among peers, regardless of their age, which allows younger people to project themselves in a more positive way, despite the limits imposed by the disease; this last point is a constitutive element of Anthony's definition of recovery [23]. iv) The meaning in life dimension was more rarely mentioned by the FG participants. We formulate the hypothesis that the group situation is perhaps less efficient on this intimate dimension, and could be favoured by individual support for life projects centred on the person's objectives. v) The empowerment dimension was one of the main dimensions reported both in terms of the role of the TPE sessions and in terms of the changes observed. Thus, all our results are in favor of the action of TPE in the recovery process according to Leamy and her colleagues. Another qualitative study carried out in our department in the same time regarding patients participating in TPE programs confirmed the benefits of the TPE program on the personal recovery of people with bipolar disorder and indicated that it improved all dimensions of recovery in the CHIME model [25]. Duval and her colleagues also stated that sharing experiences among peers promotes knowledge of the illness, a sense of being understood by others, and a sense of belonging to a group. These results are consistent, showing that patients' experience is similar to that of caregivers regarding the role and effects of the TPE program on elements of recovery in patients with bipolar disorder.

### Posture of the facilitators

The data from our study highlighted the importance of the posture of the TPE facilitator, which seems to involve continuous oscillation between being an expert (medical, experiential) and engaging in collaborative partnership aiming at horizontality. This notion of horizontality is taken up in numerous works. According to Lang [26], facilitating a session leads to.

"renouncing the omnipotence linked to one's knowledge in front of the patient, who was previously considered incompetent to manage his or her illness".

To move toward a partnership, it is necessary to build a framework of security and benevolence, as confirmed by Le Rhun [27]. Viard specified that the relationship.

"must be based on exchanges, trust and the absence of hierarchy" to move toward a partnership [28].

The recognition of the patient as a full person was discussed during our FG; according to Petré, this recognition fosters a sense of equality between the patient and the health professional as well as a more horizontal and less patriarchal relationship [29, 30]. Finally, in our study, the verbatim interview transcripts of the program facilitators emphasize the essential place of sharing experiences between peers, whether they are participants or facilitators, in the conduct of the sessions. This observation highlighted the value of the use of self-disclosure by caregivers in psychiatric care [31]. Our study showed that the facilitators respected both the scientific and national recommendations for their position as facilitators. This posture as a TPE caregiver-facilitator appeared to be shared by many authors, beyond the strict field of psychosocial rehabilitation psychiatric care. This cannot be explained by the mere fact that these professionals carried out a partial or complete part of their activity in the rehabilitation psychosocial department with a so-called "recovery-oriented practice". Indeed, according to Lang [26], the thirteen principles for a psychosocial rehabilitation practice set out by Cnaan [32] correlated with those of TPE in France: full human capacities, equipment of people with skills, self-determination, normalization, differential needs and care, commitment of staff, deprofessionalization of service, early intervention, environmental approach, changes in the environment, a lack of limits on participation, work-centered process, and social rather than medical supremacy. For Lagger, TPE promotes a change in posture and offers the opportunity to strengthen the bond, especially for the patient to be able to feel less alone with his or her chronic illness [33]. We can formulate the hypothesis that this change in posture favored by the TPE as a caregiver-facilitator could be a tool favoring a new caregiver posture as a whole and could thereby contribute through its development to psychiatric care said to be "oriented toward the recovery of people". A 2017 study explored the skills of caregivers and supports for development of recovery-supportive care. Some caregiving interventions are reported as contributing: goal setting, conversing, early intervention and anxiety management strategies; our work suggests that TPE could help in these transformations of practices and caregiving posture [34]. These results are similar to a recent review concerning the "recovery colleges" [35]. These community psycho-educational practices, open to all, have shown very similar effects on the caregivers: increased motivation to work, destigmatization of users, change in power dynamics.

To confirm this hypothesis, it would be important to carry out quantitative studies specifically focused on the effectiveness of TPE programs for people with bipolar disorder on their personal recovery.

## Limitations and future directions

Our exploratory, single-center qualitative study was conducted using an FG. This method has limitations regarding the ability of each participant to express himself or herself in a group, to express his or her own opinions and to ignore the hierarchical functional relationships between the participants, which may have led to normative responses. In particular, we noted that during this focus group, we did not hear any expression of a negative experience of this group facilitation. The choice of sampling represents another limitation: in our study, a convenience sample was used. Additionally, the FG participants belonged to the same care unit and shared the same philosophy of care. Therefore, the results may not be representative of all TPE facilitators. It would be desirable to carry out further work exploring the experiences of facilitators in a larger number of different centers; this was not easily achievable at the beginning of our study, as very few centers had implemented similar programs. To strengthen these results, it would be interesting to use qualitative methods to reach data saturation and to combine the experience of facilitators and patients. The modalities of FG facilitation could have potentially guided the participants' responses. Our study does not specify the perceived effects on participants by characteristics such as severity or duration of the disorder, or by demographic characteristics. Studies exploring quantitative or qualitative variables on these subpopulations could contribute to our knowledge.

The results of our study allow us to formulate the hypothesis that the facilitation of TPE programs could allow for a change in caregiver posture and peer-to-peer exchanges that would promote the personal recovery of people living with bipolar disorder. To confirm this hypothesis, it would also be important to carry out quantitative studies specifically focused on the effectiveness of TPE programs for the personal recovery of people with bipolar disorder. Such studies specific to TPE programs in bipolar disorder have not been published to date. There are several prerequisites for such studies, especially the choice of a relevant outcome and a suitable design. Indeed, it would be necessary to be able to precisely measure personal recovery in individuals living with bipolar disorder, as well as its determinants, to better understand how participation in the TPE program could sustain recovery. The implementation of a randomized controlled trial poses other problems in regard to psychotherapeutic actions, particularly concerning the question of the composition of the control group.

## Conclusion

The objective of our study was to explore the TPE experience from the perspective of the caregiver-facilitators. We were able to show that the posture sought by the facilitators corresponded to the national recommendations and allowed for the establishment of a particular horizontalized relationship of collaborative partnership between the facilitators and the program participants. With regards to the changes observed in the program participants by the facilitators, it appeared that they corresponded to almost all the dimensions of the CHIME recovery model, since the study found the dimensions of Connectedness, Hope, Identity and Empowerment. Further qualitative and quantitative studies would be needed to strengthen these findings.

## Data Availability

The content of the focus group analyzed in this study is available from the corresponding author on reasonable request.
